# Lipid Immunometabolism in Autoimmune Rheumatic Diseases: Mechanistic Links Between Chronic Inflammation, Lipoprotein Dysfunction and Cardiovascular Risk

**DOI:** 10.3390/biology15151270

**Published:** 2026-08-03

**Authors:** Luca Bonanni, Nicola Ferri

**Affiliations:** 1 Department of Medicine, Ospedale dell’Angelo, 30016 Venice, Italy; 2 Department of Medicine, University of Padua, 35131 Padua, Italy; nicola.ferri@unipd.it

**Keywords:** lipid immunometabolism, autoimmune rheumatic diseases, cardiovascular risk, lipid paradox, lipoprotein dysfunction, HDL dysfunction, oxidized LDL, macrophage cholesterol metabolism, NLRP3 inflammasome, thrombo-inflammation

## Abstract

Patients with autoimmune rheumatic diseases, particularly rheumatoid arthritis (RA) and systemic lupus erythematosus (SLE), experience excess cardiovascular risk that is not fully captured by conventional lipid measurements. In active RA, lower cholesterol may coexist with higher vascular risk, a pattern known as the lipid paradox. We propose that systemic inflammation can uncouple lipid concentration from lipoprotein function and organize the evidence along five mechanistic axes. Inflammatory cytokines, mainly interleukin-6 (IL-6), tumor necrosis factor-alpha (TNF-α), IL-1β, IL-17/IL-23 and type I interferons, remodel lipoprotein metabolism. High-density lipoproteins (HDL) lose protective functions and may become pro-inflammatory. Apolipoprotein-B particles are oxidized or otherwise modified, linking lipid metabolism to autoimmunity. Macrophage cholesterol imbalance and cholesterol crystals activate inflammasome pathways in experimental atherosclerosis, while immune-cell metabolic rewiring may amplify cytokine output; these mechanisms are treated as extrapolated when direct rheumatic-disease evidence is limited. The pathways converge on endothelial dysfunction and thrombo-inflammation. RA and SLE are the mechanistic anchors, whereas psoriatic disease, axial spondyloarthritis, systemic sclerosis, vasculitides and antiphospholipid syndrome are weighted by evidence category. Standard lipid panels may therefore underestimate risk in selected contexts, especially during active inflammatory disease.

## Simple Summary

People with autoimmune rheumatic diseases, such as rheumatoid arthritis and lupus, have a higher chance of heart attacks and strokes than their cholesterol levels alone would predict. This is especially striking in active rheumatoid arthritis, where cholesterol often falls while vascular risk remains high. This review explains why a normal lipid panel can be misleading in this setting. Long-lasting inflammation changes not only the amount of fat carried in the blood, but also the quality and behavior of the particles that carry it. Protective particles may lose their normal functions, other particles may become chemically altered and immunogenic, and immune cells may accumulate cholesterol inside the vessel wall and amplify inflammation. These processes can damage the endothelium and favor thrombosis. Rheumatoid arthritis and systemic lupus erythematosus provide the strongest direct evidence; other rheumatic diseases are discussed according to the strength and type of available data. This framework supports a combined preventive strategy in which inflammatory disease control and lipid management are considered together, while recognizing that several proposed mechanisms remain extrapolated from broader atherosclerosis or experimental research.

## 1. Introduction

Autoimmune rheumatic diseases are associated with excess mortality, to which cardiovascular events make a major contribution, most clearly in rheumatoid arthritis (RA), and the excess risk is only partly explained by the conventional risk factors that these patients accumulate [1,2]. In RA, the discordance with the lipid profile is striking. During periods of high disease activity, total and low-density lipoprotein cholesterol (LDL-C) tend to fall, while cardiovascular risk remains elevated, so that lower LDL-C is associated at the population level with higher rather than lower risk. This inversion of the expected relationship between cholesterol and risk is the lipid paradox, and it was demonstrated directly in a population-based incident cohort in which low total and LDL-C marked higher cardiovascular risk while inflammatory activity tracked with events [1,3,4]. Similar dissociations between lipid concentration and vascular outcome have been reported or inferred in other autoimmune rheumatic diseases, but the strength and type of evidence vary substantially by condition.

The depth of evidence is uneven across these conditions. RA and SLE provide the deepest direct human mechanistic and clinical evidence for lipoprotein remodeling, HDL dysfunction, modified lipoproteins and vascular injury. In psoriatic disease, axial spondyloarthritis, systemic sclerosis, vasculitis and antiphospholipid syndrome, the model is supported by different combinations of observational outcome data, imaging or surrogate studies, disease-specific mechanisms and inference from general atherosclerosis research. The aim of the present review is therefore not to claim that one mechanism is equally proven across all diseases, but to map how chronic inflammation, lipid and lipoprotein remodeling and vascular injury may be connected, and to identify where conventional lipid testing may underestimate risk, particularly during active inflammatory disease.

The paradox becomes tractable once atherosclerosis is considered as an inflammatory process rather than a purely lipid-storage one. Studies conducted in the general population show that inflammatory risk persists after lipids are aggressively lowered. In statin-treated and statin-intolerant cohorts, high-sensitivity C-reactive protein (hsCRP) and interleukin-6 (IL-6) predict recurrent events at least as strongly as LDL-C, and in some strata even more strongly [5,6]. Autoimmune rheumatic diseases may be regarded as chronic amplifiers of this residual inflammatory risk, maintained over years and driven by disease-specific cytokine programs.

Previous reviews have treated the lipid paradox in RA, HDL dysfunction in SLE, cardiovascular risk across autoimmune rheumatic diseases, and the effects of antirheumatic drugs on lipids as largely separate topics. The argument here connects them into a single mechanistic circuit: chronic inflammation remodels lipoprotein function, functionally altered lipoproteins reshape the behavior of immune cells, macrophage cholesterol handling activates inflammasome signaling, and endothelial and thrombo-inflammatory pathways convert these events into vascular injury. Two further elements distinguish the account. It grades the strength of evidence disease by disease rather than assuming that mechanisms established in RA or lupus apply unchanged to every condition, and it makes the immunometabolic layer, including trained immunity, explicit as the cellular substrate on which the lipoprotein changes act.

**Figure 1:**
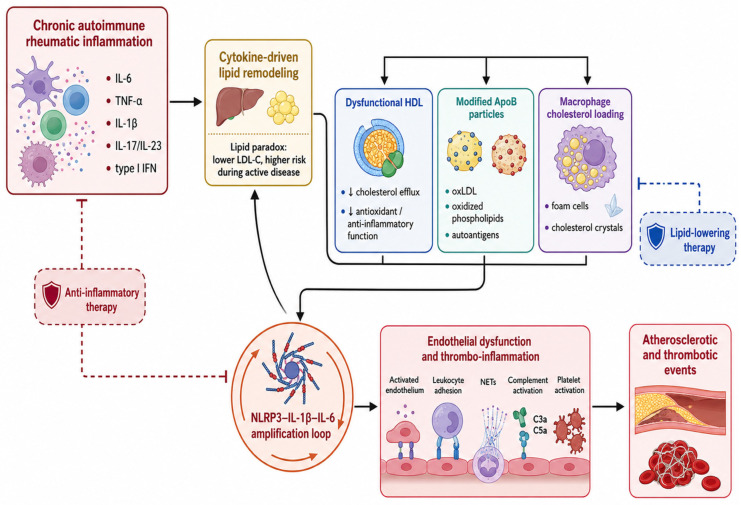
Integrated circuit linking chronic autoimmune rheumatic inflammation to cardiovascular events. The figure is a conceptual synthesis rather than a quantitative causal diagram. Pathways best supported by direct human rheumatic-disease evidence are interpreted as stronger links, whereas pathways based mainly on experimental atherosclerosis, general-population cardiovascular evidence or mechanistic inference are indicated in the legend and treated more cautiously in the text. Chronic inflammation drives cytokine-mediated lipid remodeling (IL-6, interleukin-6; TNF-α, tumor necrosis factor-alpha; IL-1β, interleukin-1beta; IL-17/IL-23, interleukin-17/interleukin-23; type I IFN, type I interferon), producing a divergence between lipid concentration and lipoprotein function that is clinically visible as the lipid paradox, especially in active RA. Three lipoprotein-level processes follow in parallel: conversion of HDL (high-density lipoprotein) into a dysfunctional, sometimes pro-inflammatory particle with reduced cholesterol efflux capacity; oxidation and modification of ApoB (apolipoprotein-B) particles, which generate autoantigens; and macrophage cholesterol loading with foam-cell formation, supported by a metabolically rewired immune compartment. Cholesterol crystal-NLRP3 (NLR family pyrin domain containing 3) activation and trained immunity are mainly supported by experimental or general atherosclerosis evidence and are therefore shown as extrapolated components when applied to rheumatic-disease vessels. The streams converge on a dysfunctional, thrombo-inflammatory endothelium, yielding atherosclerotic and thrombotic events. Anti-inflammatory and lipid-lowering therapies intercept different parts of the circuit.

This review focuses on how systemic inflammation in autoimmune rheumatic diseases reshapes lipid and lipoprotein biology so that cardiovascular risk can become partly detached from the lipid concentrations measured in routine clinical practice. Although the motivating problem is clinical, the review is built around cell– and molecular–biological mechanisms: macrophage cholesterol handling, inflammasome activation, lipoprotein biology, endothelial function and immune-cell metabolism. The sections that follow assemble a causal architecture rather than a list of associations: five mechanistic axes that move from the initiating cytokine signal to the vessel wall, followed by disease-specific weighting, a therapeutic stress test of the model, and the implications for risk stratification (Figure 1).

## 2. Literature Search and Conceptual Framework

This is a mechanistic narrative review, not a systematic review; no PRISMA protocol was applied and no claim is made to systematic completeness. The final targeted literature search for the revised manuscript was performed on 24 July 2026. The literature was identified through PubMed/MEDLINE, supplemented by Scopus and Web of Science and by complementary citation searching of key articles and reviews. Representative search strings combined disease terms with mechanistic and clinical terms, for example: (rheumatoid arthritis OR systemic lupus erythematosus OR psoriatic arthritis OR axial spondyloarthritis OR systemic sclerosis OR vasculitis OR antiphospholipid syndrome) AND (cardiovascular risk OR atherosclerosis OR lipid paradox OR HDL dysfunction OR cholesterol efflux OR oxidized LDL OR lipoprotein(a) OR NLRP3 OR inflammasome OR endothelial dysfunction OR thrombo-inflammation). Treatment-focused searches combined the same disease terms with methotrexate, hydroxychloroquine, glucocorticoids, TNF inhibitors, IL-6 receptor inhibitors, JAK inhibitors, IL-17/IL-23 inhibitors, statins, ezetimibe and PCSK9 inhibitors.

The time window was preferentially 2010 to 2026, with older seminal mechanistic and experimental studies included where they remain foundational. Priority was given to human rheumatic-disease outcome studies, human rheumatic-disease mechanistic studies, vascular imaging and surrogate studies, meta-analyses, randomized trials where available, and guideline documents. Experimental papers and general-population cardiovascular studies were retained when they established causal or mechanistic links that cannot be directly tested in human rheumatic-disease tissue. Studies were de-emphasized when they were purely descriptive, when the patient population was not clearly defined, or when the mechanistic link to lipid immunometabolism was indirect and not clinically or biologically informative. When findings conflicted, human rheumatic-disease data were prioritized over extrapolated evidence, and evidence limitations were stated explicitly rather than resolved by narrative preference.

For consistency across the text and tables, evidence was assigned to six pragmatic categories: (1) human rheumatic-disease outcome evidence; (2) human rheumatic-disease mechanistic evidence; (3) human surrogate or imaging evidence; (4) general-population cardiovascular genetic, biomarker or trial evidence; (5) experimental or preclinical atherosclerosis evidence; and (6) mechanistic inference or extrapolation. These categories are not intended as a formal GRADE assessment, but as a transparency framework for distinguishing mechanisms directly shown in rheumatic diseases from those borrowed from broader cardiovascular biology. They are applied in the disease-specific table, the molecular mediator table and the figure legends, particularly for trained immunity, cholesterol crystal-NLRP3 activation, NET-mediated lipoprotein oxidation and proposed feedback loops.

## 3. Cytokine-Driven Remodeling of Lipid Metabolism

The first axis concerns how inflammatory mediators act on lipid handling. IL-6, TNF-α, IL-1β, the IL-17/IL-23 axis and type I interferon each influence hepatic lipoprotein production, peripheral clearance and the enzymatic machinery that determines particle composition [2,7]. The hepatic acute-phase response redirects lipid metabolism toward host defense, which alters cholesterol synthesis and catabolism and modifies the proteins carried on lipoprotein particles. A practical consequence follows: the concentration measured in plasma and the function of the particle can move in opposite directions.

IL-6 represents a key inflammatory mediator linking cytokine signaling to lipid remodeling. Genetic and pharmacological data place IL-6 signaling on a causal pathway for coronary disease in the general population, and IL-6 also mediates hepatic acute-phase responses that reshape circulating lipoproteins [5,8]. The clearest rheumatic-disease mechanistic window comes from IL-6 receptor (IL-6R) blockade. In a randomized study of patients with active RA, tocilizumab raised total cholesterol, LDL-C and triglycerides, yet several qualitative markers shifted toward a less atherogenic profile: HDL serum amyloid A content fell, paraoxonase activity rose, and lipoprotein(a) [Lp(a)], secretory phospholipase A2-IIA, fibrinogen and D-dimer all decreased [9]. The rise in cholesterol concentration coincided with an apparent improvement in particle quality and in coagulation surrogates. Whether the net effect favors or harms the vessel is not resolved by concentration alone, which is the problem the rest of this review addresses.

Lp(a) illustrates the same point from a different angle. Its plasma level is largely genetically set, but it behaves in part as an inflammatory particle, and elevated levels are observed in RA, SLE and chronic renal inflammation [10]. IL-6R blockade lowers lipoprotein(a) in RA, which indicates that inflammatory signaling can modulate even a strongly heritable lipoprotein [8,9]. Reductions in inflammation therefore do more than change a number on a lipid panel. They reorganize the particles themselves.

A reasonable reading of these data is that suppression of inflammation does not simply normalize lipid levels. It can unmask a cardiometabolic profile that active disease had concealed, raising LDL-C while removing the inflammatory drive that made a lower LDL-C dangerous [7]. The concentration change and the risk change are governed by partly separate processes. This separation is the mechanistic root of the lipid paradox, and the axes that follow describe where, at the level of the particle and the cell, that separation is generated.

## 4. Alternative Explanations and Causal-Inference Evidence for the Lipid Paradox

The lipid paradox should not be read as evidence that low LDL-C is itself a cause of cardiovascular events. The inverse association between cholesterol concentration and risk in active RA has several plausible contributors that operate alongside, or instead of, a direct causal effect. Systemic inflammation lowers measured total and LDL-C, so a low value can be a marker of high inflammatory activity rather than a protective state; in a population-based incident cohort, low total cholesterol identified patients at higher cardiovascular risk while inflammatory activity, indexed by the erythrocyte sedimentation rate, was itself associated with events [4]. Reverse causation of this kind is compounded by altered body composition and inflammatory cachexia, by frailty in long-standing disease, by indication bias in who receives statins, by confounding from disease severity and glucocorticoid exposure, and by changes in treatment era that alter both inflammation and lipid management. Each of these can generate an inverse lipid–risk association without any need for low cholesterol to be causal.

These considerations qualify the paradox without dissolving the mechanistic model. What they establish is that lipid concentration is context-dependent: the same number means different things at different levels of inflammation, because inflammation simultaneously lowers plasma cholesterol levels and degrades the function of the particles that carry it. The model does not claim that low LDL-C causes events; it claims that conventional concentration measurements lose their usual meaning when lipoprotein quality is altered. Human causal-inference data from the general population support the inflammatory half of this argument. Genetic variation in the IL-6R that mimics receptor blockade is associated with lower CRP and a modestly lower risk of coronary heart disease, which places IL-6 signaling on the causal path to vascular disease rather than beside it [8]. Trials of pathway-specific anti-inflammatory therapy point the same way: neutralization of IL-1β reduced recurrent events without lowering lipids, whereas low-dose methotrexate, which did not reduce IL-6 or CRP in a non-rheumatic population, did not [11,12]. Genetic data also confirm that a strongly heritable, inflammation-responsive lipoprotein, Lp(a), is causally related to myocardial infarction [13]. These findings are supportive of biological context. They derive from general-population genetics and trials, not from rheumatic-disease cohorts, and they should be read as strengthening the plausibility of the inflammatory mechanism rather than as proof that the same quantitative relationships hold in autoimmune rheumatic disease.

## 5. HDL Dysfunction and Loss of Atheroprotection

HDL is inversely associated with cardiovascular risk in the general population because of functions it performs rather than the cholesterol it contains: cholesterol efflux, antioxidant activity through paraoxonase, preservation of endothelial homeostasis and restraint of vascular inflammation [14]. Drugs that raise HDL-cholesterol without restoring these functions have not reduced events, which already shows that concentration and function are dissociable [14]. Inflammation drives that dissociation. Under an acute-phase response the HDL proteome and lipidome are remodeled, with enrichment of serum amyloid A and loss or inactivation of protective enzymes, producing a particle that performs its tasks poorly and may itself become pro-inflammatory [14,15]. This shift from atheroprotective to dysfunctional HDL is a defining element of lipoprotein dysfunction in inflammatory disease.

In autoimmune rheumatic diseases the conversion is well documented. In SLE, systemic inflammation, oxidative stress and autoimmunity change HDL size distribution and its proteomic and lipidomic signatures, generating dysfunctional HDL with reduced cholesterol efflux capacity, impaired antioxidant activity and diminished anti-inflammatory function; such HDL can promote rather than restrain atherogenesis [16]. In women with SLE, this pro-inflammatory HDL phenotype is common and is strongly associated with subclinical atherosclerosis, with a large increase in the odds of carotid plaque, reduced paraoxonase-1 activity and modification of apolipoprotein A-I [17]. A parallel literature in RA and across rheumatic conditions describes the same loss of lipoprotein function, with patient serum showing an altered capacity to handle cellular cholesterol [15]. In RA, the anti-inflammatory function of HDL falls as disease activity rises, and dysfunctional HDL carries an altered protein cargo, with enrichment of serum amyloid A and myeloperoxidase, reduced lecithin:cholesterol acyltransferase (LCAT) activity and modified apolipoprotein A-I (apoA-I); serum amyloid A can displace apoA-I from the particle, so an unchanged HDL-cholesterol value can accompany a functionally remodeled HDL [18]. The protective particle is not merely reduced in quantity. It is functionally subverted, and the subversion has consequences beyond efflux, because functional HDL normally restrains Toll-like receptor (TLR) signaling and supports cholesterol efflux from immune cells, a brake that is lost when HDL becomes dysfunctional [19].

Function, when measured directly, carries prognostic weight. Cholesterol efflux capacity is inversely associated with cardiovascular events: a meta-analysis of twenty cohorts found that higher efflux capacity predicted fewer events independently of HDL-cholesterol and traditional risk factors [20], a result anticipated by a population cohort in which efflux capacity, but not HDL-cholesterol concentration, predicted incident cardiovascular events [21]. The relationship is not uniform across vascular beds. In a population study, higher cholesterol mass efflux capacity tracked with lower incident coronary heart disease but, paradoxically, with greater carotid plaque progression, which cautions against treating any single functional readout as a universal protective marker [22]. For autoimmune rheumatic diseases, the implication is that HDL-cholesterol concentration may appear reassuring while the particle has lost functions that make HDL protective, especially during active inflammation. This is one reason why conventional lipid assessment may underestimate risk in selected patients.

## 6. Modified Lipoproteins at the Interface of Oxidation, Autoimmunity and Vascular Injury

The third axis links lipid metabolism to autoimmunity through chemical modification of lipoproteins. Oxidation is the prototype. Oxidized LDL (oxLDL) is taken up by macrophage scavenger receptors, contributes to foam-cell formation, and carries neoepitopes that the adaptive immune system recognizes [23]. In autoimmune disease this recognition is amplified. Antibodies against oxLDL, against β2-glycoprotein I, and against oxLDL–β2GPI complexes have been described in SLE and APS, and a defective clearance of dead cells and modified particles is a feature shared by atherosclerosis and lupus [23,24]. Circulating oxLDL forms stable, nondissociable complexes with β2-glycoprotein I, and high serum levels of these complexes, together with IgG antibodies that recognize them, are associated with arterial thrombosis in APS, which makes the oxLDL–β2GPI complex a disease-specific bridge between lipoprotein oxidation and atherothrombosis [25]. The same particle therefore operates as a metabolic substrate, a danger signal and an autoantigen.

The relationship between these antibodies and outcome is not monotonic, which is mechanistically informative. Some natural antibodies, such as those against phosphorylcholine, are associated with less atherosclerosis and may be protective, whereas antiphospholipid antibodies associate with both arterial and venous events [23]. The autoimmune response to modified lipids can thus act in opposing directions depending on specificity, and this heterogeneity is one reason that single antibody measurements have limited predictive value. Beyond oxidation, lipoproteins in rheumatic disease undergo additional post-translational modifications, including carbamylation, that generate further neoepitopes and may sustain autoantibody responses; the direct evidence linking carbamylated lipoproteins to events in rheumatic populations is fragmentary and should be read as suggestive rather than settled [15].

Neutrophils tie modification, autoimmunity and the vessel together. Neutrophil extracellular traps (NETs) are increased in SLE and RA and carry oxidizing enzymes, such as myeloperoxidase, externalize autoantigens, and prime inflammatory responses [26]. In RA, NETs externalize citrullinated autoantigens, are induced by IL-17A and TNF-α, and stimulate synovial fibroblasts to release IL-6 and IL-8 [27]. In SLE, a low-density granulocyte subset forms NETs that damage endothelial cells and expose immunostimulatory material, linking aberrant neutrophil biology directly to vascular injury [28]. NETs are increasingly implicated in atherosclerosis and may contribute to the premature vascular disease seen in autoimmunity, both by activating endothelial and immune cells and, through the myeloperoxidase (MPO) they carry, by potentially oxidizing lipoproteins in the local vascular environment; this last step is inferred from the oxidizing activity of NET-associated enzymes rather than directly demonstrated in rheumatic-disease vasculature [26]. In experimental atherosclerosis, cholesterol crystals trigger NETs that prime macrophages for cytokine production and activate T helper 17 responses, connecting crystal formation to neutrophil-driven amplification [29]. A feed-forward loop is plausible: modified lipoproteins and NET components each amplify the other and each supply antigenic material that perpetuates the autoimmune response. The quantitative contribution of this loop to clinical events in patients remains to be established, and current evidence is largely mechanistic.

## 7. Macrophage Cholesterol Handling, Foam Cells and the NLRP3–IL-1β–IL-6 Circuit

The fourth axis moves inside the artery wall to the macrophage, and it is here that lipid handling and innate immune activation become inseparable. Atherogenesis depends on the balance between uptake of modified LDL, mainly through scavenger-receptors such as CD36, scavenger receptor-A and LOX-1, and cholesterol efflux through the transporters ABCA1 and ABCG1 onto functional HDL [15,19]. SR-BI has a distinct and more complex role in HDL-associated lipid transport and bidirectional cholesterol flux, and should not be considered equivalent to CD36 or LOX-1 as a modified-LDL uptake pathway. Inflammation tips the macrophage balance in both directions at once. Efflux through ABCA1 and ABCG1 is transcriptionally controlled by the liver X receptors (LXR), which sense sterol load and induce the transporters, while TLR signaling opposes this program; inflammatory activation therefore lowers efflux at the level of gene expression, even as uptake of modified particles continues. Cholesterol then accumulates in macrophages and other vascular-wall cells, and macrophages become foam cells [15,19,30]. Cholesterol efflux is not only a lipid-handling pathway but an immunological brake: in myeloid cells, intact ABCA1 and ABCG1 function restrains NLRP3 inflammasome activation and neutrophil extracellular trap formation, and its loss raises IL-1β and IL-18 release, a connection confirmed in people carrying loss-of-function ABCA1 mutations [31]. The reverse coupling also holds: cholesterol accumulation augments TLR and inflammasome signaling, which establishes a self-reinforcing relationship between cholesterol loading and inflammation at the level of the single cell [19]. In APS, the oxLDL-β2GPI complex itself drives macrophage differentiation into foam cells, which connects a disease-specific autoantibody target directly to lipid loading [25,32].

Accumulated cholesterol is not inert. Once it crystallizes, it becomes an endogenous danger signal. Inflammasome output requires two signals. A priming signal, typically through Toll-like receptor and NF-κB activation, induces NLRP3 and pro-IL-1β; an activation signal, supplied by cholesterol crystals that destabilize the phagolysosome, triggers assembly of the inflammasome and caspase-1-dependent cleavage and secretion of IL-1 family cytokines [33,34]. The scavenger-receptor CD36 couples the two steps: by internalizing soluble oxLDL and nucleating it into cholesterol crystals inside the cell, CD36 converts a soluble ligand into the particulate trigger that the inflammasome senses [35]. Murine work established that NLRP3 inflammasome components are required for diet-induced atherogenesis and that cholesterol crystals appear early, coincident with the first inflammatory cells, which reframed them as an initiating rather than a terminal feature of the plaque [33]. In this pathway, IL-1β induces IL-6 production mainly in immune, endothelial and stromal compartments, while IL-6 acts on hepatocytes to drive the acute-phase response and associated lipoprotein remodeling [5,11,36]. Much of the supporting mechanism derives from experimental atherosclerosis; direct demonstration of cholesterol-crystal-driven NLRP3 activation within rheumatic-disease tissue is more limited, so the pathway is best regarded as strongly supported in atherogenesis and plausibly operative, rather than fully proven, in the rheumatic-disease vessel.

**Figure 2:**
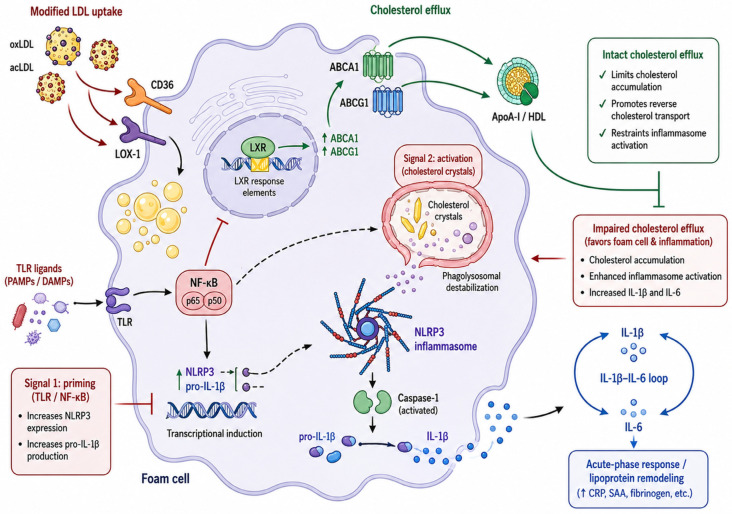
Molecular detail of macrophage cholesterol handling and NLRP3 inflammasome activation. Modified LDL (low-density lipoprotein) is taken up mainly through CD36 (cluster of differentiation 36) and LOX-1 (lectin-like oxidized LDL receptor-1), whereas SR-BI (scavenger-receptor class B type I) has a distinct role in HDL-associated lipid transport and bidirectional cholesterol flux and is therefore not depicted as equivalent to CD36 or LOX-1. Cholesterol efflux to ApoA-I (apolipoprotein A-I) and HDL (high-density lipoprotein) proceeds through ABCA1 and ABCG1 (ATP-binding cassette transporters A1 and G1), whose expression is induced by LXR (liver X receptors) and opposed by TLR (Toll-like receptor) and NF-κB (nuclear factor kappa B) signaling. When uptake exceeds efflux, free cholesterol accumulates and crystallizes. A priming signal (signal 1) through TLR and NF-κB activation induces NLRP3 and pro-IL-1β; an activation signal (signal 2) is supplied by cholesterol crystals that destabilize the phagolysosome, with CD36 nucleating soluble oxLDL into intracellular crystals. The assembled inflammasome activates caspase-1, which cleaves pro-IL-1β to mature IL-1β. IL-1β promotes IL-6 production mainly in immune, endothelial and stromal compartments, while IL-6 acts on hepatocytes to sustain the acute-phase response and lipoprotein remodeling. Intact ABCA1- and ABCG1-mediated efflux restrains inflammasome activation, so loss of efflux amplifies the loop. The pathway is strongly supported in experimental atherosclerosis and is plausibly operative in the rheumatic-disease vessel, where direct tissue demonstration remains more limited.

Reading the cytokine axis and the macrophage axis together exposes a circuit rather than a linear chain. Cholesterol that the inflamed system handles poorly can crystallize and activate NLRP3; the resulting IL-1β promotes IL-6 production in local immune and stromal compartments; IL-6 then sustains hepatic acute-phase responses and lipoprotein remodeling, while also supporting further immune activation. The output of the cytokine axis therefore becomes an input that may perpetuate it. In autoimmune rheumatic disease, where systemic inflammatory drive can persist for years, this feedback loop is biologically plausible, but its quantitative contribution to clinical events remains incompletely defined (Figure 2).

## 8. Immune-Cell Metabolic Rewiring as the Cellular Substrate of Lipid-Driven Inflammation

The preceding axes describe what happens to lipoproteins and to cholesterol handling. They rest on a cellular substrate that deserves explicit treatment, because the metabolic state of immune cells determines how strongly they respond to lipid signals and how much cytokine they produce. This axis is mechanistically well supported at the cellular level, while its direct contribution to cardiovascular risk prediction in patients remains largely inferential, a distinction that should be kept in view throughout.

In RA, naive CD4 T cells carry a disease-specific metabolic signature. Glucose is shunted away from glycolytic breakdown toward the pentose phosphate pathway, favoring anabolic over catabolic reactions, with downstream consequences that include mitochondrial DNA instability and deficient lysosomal tethering of AMP-activated protein kinase, the latter releasing a brake on mechanistic target of rapamycin signaling [37]. These metabolically reprogrammed T cells differentiate into pro-inflammatory effectors that invade tissue and elicit inflammation, which links cellular energy handling to the cytokine program that drives lipid remodeling [37]. The same study frames macrophage metabolic state as part of the disease process rather than a bystander.

Macrophage metabolism completes the connection to lipids. Cholesterol accumulation and TLR activation reshape macrophage metabolic programs, and the resulting cells couple impaired cholesterol efflux to heightened inflammatory output [19]. Beyond acute activation, myeloid cells retain a durable hyperresponsive state after exposure to metabolic danger signals, a phenomenon termed trained immunity. Western-type dietary stimulation induces NLRP3-dependent epigenetic and metabolic reprogramming of myeloid progenitors, producing monocytes with augmented inflammatory responses that persist after the stimulus is withdrawn, and oxLDL is among the endogenous inducers of this state [38]. The oxLDL effect has been shown directly at the molecular level: brief exposure of human monocytes to oxLDL produces a durable pro-inflammatory phenotype with increased CD36 and scavenger-receptor-A, decreased ABCA1 and ABCG1, and trimethylation of histone H3 at lysine 4 across pro-inflammatory and scavenger-receptor gene promoters [39]. Trained immunity is maintained by epigenetic remodeling together with metabolic shifts in glycolysis, glutaminolysis and the cholesterol–mevalonate pathway, and in the setting of chronic systemic inflammation, it appears maladaptive, sustaining vascular inflammation rather than resolving it [40].

Two qualifications matter. First, immune-cell metabolic rewiring is mechanistically well supported in RA, and trained immunity is well supported in atherosclerosis and innate immune biology, but direct evidence linking trained immunity to cardiovascular events in rheumatic-disease cohorts remains limited. In autoimmune rheumatic diseases, this trained-immunity framework therefore remains mainly extrapolative, although it is biologically coherent with persistent systemic inflammation and repeated exposure to endogenous danger signals. Second, acute metabolic activation and durable epigenetic reprogramming are related but not identical. The former describes short-term changes in cellular fuel use during activation, whereas trained immunity implies persistent chromatin and metabolic remodeling that changes the response to later stimuli. The functional importance of this axis for the present argument is conceptual: dysfunctional HDL loses its capacity to limit immune-cell cholesterol loading and inflammatory signaling, so the lipoprotein dysfunction of axes two and three removes a metabolic brake, and the immunometabolic state of the cell amplifies the cytokine output that returns to remodel lipids. The cellular and the lipoprotein layers are therefore two levels of one circuit, but the clinical predictive value of this layer remains unvalidated.

## 9. Endothelial Dysfunction and Thrombo-Inflammation as the Convergent Endpoint

The preceding mechanisms converge on the endothelium, the point at which lipid immunometabolism becomes a vascular event. Modified lipoproteins reduce nitric oxide (NO) bioavailability and activate endothelial cells; the resulting expression of adhesion molecules recruits leukocytes and supports a procoagulant surface. In autoimmune disease, additional drivers act in parallel. Type I interferon promotes endothelial dysfunction both directly and by priming immune cells, and aberrant neutrophils injure the vessel through NET formation [28,41]. The endothelium in SLE is therefore damaged by an immune program that operates largely independently of conventional risk factors, which helps explain why those factors fail to account for the observed risk [41].

Thrombo-inflammation links this endothelial injury to clinical events. In APS, antiphospholipid antibodies activate endothelial cells and monocytes, engage complement and neutrophils, promote NET formation, and drive platelet activation and thrombin generation, in parallel with oxLDL–β2GPI-induced foam-cell formation [32]. The antibody profile that causes thrombosis therefore also accelerates atherosclerosis, and the two processes share effectors. This convergence suggests that thrombo-inflammation is not a separate complication added to atherosclerosis but a final common pathway through which lipid modification, immune activation and coagulation produce arterial and venous events together. The functional vascular consequences are measurable as impaired flow-mediated dilation, increased arterial stiffness and greater carotid intima–media thickness, abnormalities demonstrable across rheumatic diseases including the spondyloarthritides [42] and systemic sclerosis [43].

## 10. Disease-Specific Weighting of Shared Mechanisms

Across the conditions that follow, RA and SLE are the mechanistic anchors, because they carry the deepest combination of human outcome and human mechanistic evidence for the axes described above [4,17]. The remaining diseases are discussed as comparators and extensions, in which the same circuit is weighted differently and is supported by evidence of variable strength, from observational and imaging data to disease-specific mechanisms or extrapolated inference. The aim is to show where the shared framework applies with confidence and where it remains provisional.

Table 1 summarizes the dominant lipid-immune mechanism, principal mediators, characteristic vascular phenotype and evidence category by disease. The categories correspond to the framework defined above and separate human rheumatic-disease outcome evidence, human mechanistic evidence, surrogate or imaging evidence, general-population evidence, experimental evidence and mechanistic inference. This distinction is clinically important because it determines whether a proposed mechanism should be treated as a demonstrated driver of risk, a plausible biological contributor, or a hypothesis requiring direct testing.

In RA, the excess risk is best explained by the cytokine-driven combination of qualitative lipoprotein remodeling, the lipid paradox during active disease, impaired HDL function and accelerated coronary atherosclerosis, with disease activity itself behaving as a vascular risk factor; this is supported by human outcome and human mechanistic data [1,2,7]. Immunometabolic rewiring of T cells and macrophages provides an upstream cellular substrate for the chronic activation [37].

In SLE, type I interferon, immune complexes, complement, antiphospholipid antibodies, dysfunctional HDL and oxLDL act together to produce both accelerated atherosclerosis and thrombosis [16,23,41]. Lupus shows most clearly that vascular injury can be driven by an immune program rather than by lipid concentration, with strong human mechanistic evidence and moderate outcome data.

In psoriatic disease, IL-17/IL-23-driven inflammation coexists with a high prevalence of metabolic syndrome, insulin resistance, obesity and triglyceride-rich dyslipidemia, and these cardiometabolic comorbidities track with disease extent and influence treatment response [44,45]. The lipid abnormality here is more overtly atherogenic in the conventional sense than in RA, while still being amplified by inflammation, and the evidence base combines observational outcome data with surrogate markers.

In ankylosing spondylitis and axial spondyloarthritis, subclinical atherosclerosis is consistently demonstrable: meta-analysis shows increased carotid intima–media thickness and arterial stiffness and reduced flow-mediated dilation compared with controls, evidence that is predominantly imaging-based [42].

In systemic sclerosis, microvascular disease is the central and defining vascular abnormality, and it should be distinguished from macrovascular atherosclerosis. Macrovascular involvement, accelerated atherosclerosis and increased arterial stiffness have been reported across several methods, but the evidence is heterogeneous and debated, rests largely on surrogate measures, and does not establish the lipid-immunometabolic model to the degree reached in RA or SLE; endothelial dysfunction and oxidative stress provide its most plausible mechanistic link [43].

The vasculitides warrant separation from antiphospholipid syndrome rather than grouping with it, because their mechanisms differ. In the large-vessel vasculitides, giant cell arteritis and Takayasu arteritis, ischemic heart disease, cerebrovascular disease and aortic disease are increased, and the relative contributions of active vasculitis, vascular damage and accelerated atherosclerosis remain debated [46]. In ANCA-associated vasculitis, a population-based cohort found increased rates of stroke, atrial fibrillation and heart failure relative to matched controls, particularly in the period soon after diagnosis, although the composite of major adverse cardiovascular events was not significantly increased [47]; endothelial dysfunction is described, and the endothelial response may differ mechanistically from classical atherosclerosis, which cautions against assuming an identical lipid-driven process [48]. Across the vasculitides, vascular inflammation and endothelial injury are central, whereas a specific lipid-immunometabolic contribution is largely inferred rather than directly demonstrated.

Antiphospholipid syndrome is best read as a disease-specific model of thrombo-inflammation and atherothrombosis. The oxLDL–β2GPI complex and antiphospholipid antibodies are mechanisms particular to this syndrome and are not generalizable to the other rheumatic diseases; the oxidized lipoprotein and the prothrombotic autoantibody act together to produce arterial and venous events, supported by mechanistic and observational data [32]. Guideline recommendations across both the vasculitides and APS are frequently based on limited evidence [49].

## 11. Therapeutic Modulation as a Clinical Stress Test of the Model

Therapy provides a way to test the proposed hierarchy under real-world conditions. If lipid concentration alone were the main driver of risk, then a treatment-induced rise in LDL-C would necessarily imply increased risk. The data do not behave that way, and the pattern of exceptions is itself informative. Table 2 sets out the effects of the main agents on lipid concentration, on lipoprotein function or inflammation where known, the net cardiovascular signal, and the principal interpretative caveat for each.

IL-6R blockade is the cleanest test. Tocilizumab raises LDL-C while shifting HDL composition toward an anti-inflammatory profile and lowering Lp(a), fibrinogen and D-dimer, so the lipid concentration moves in the apparently adverse direction while several functional and thrombotic surrogates improve [8,9]. A randomized cardiovascular outcome trial sharpens the point: in RA patients with at least one cardiovascular risk factor, tocilizumab raised LDL-C, HDL-C and triglycerides relative to etanercept by week 4, yet the major adverse cardiovascular event hazard ratio for tocilizumab vs. etanercept was approximately 1.05 (95% confidence interval roughly 0.77–1.43) [50]. The upper bound of this interval lay below the trial’s prespecified non-inferiority margin, so a large excess of events was excluded and non-inferiority to etanercept was met, but cardioprotection was not demonstrated. The honest interpretation is that this dissociation argues against concentration as the sole driver of risk, without establishing that tocilizumab is cardioprotective, because the net vascular effect of these opposing changes is not resolved by a trial powered to exclude a large harm rather than to prove benefit. Conventional synthetic and biologic disease-modifying drugs that control disease activity tend to improve the cardiovascular outlook, and the effect appears to track with inflammation control rather than with any particular change in the lipid panel [2,7]. Hydroxychloroquine illustrates a complementary, lipid-favorable mechanism: its use is associated with fewer incident cardiovascular events, described most clearly in rheumatoid arthritis cohorts, alongside a less atherogenic lipid profile and antithrombotic properties [51]. TNF-α inhibitors generally reduce vascular inflammation and are associated with favorable cardiovascular signals, and a meta-analysis reported lower cardiovascular event rates with TNF-α inhibitors and with methotrexate, and higher rates with corticosteroids, in RA and psoriatic disease, although patient selection and the confounding effect of disease control complicate causal attribution, and TNF-α inhibitors can promote weight gain [45,52].

The Janus kinase inhibitors are the decisive counterexample, and they prevent any simple equation of inflammation control with cardiovascular protection. In ORAL Surveillance, a randomized safety trial enriched for cardiovascular risk, tofacitinib did not meet non-inferiority against TNF-α inhibition for major adverse cardiovascular events (3.4% vs. 2.5%; hazard ratio 1.33, 95% confidence interval 0.91–1.94) and cancers (4.2% vs. 2.9%; hazard ratio 1.48, 95% confidence interval 1.04–2.09), despite comparable control of disease activity [53]. JAK inhibitors also raise lipid levels. This result anchors current guidance, which recommends caution with these agents in patients with cardiovascular risk factors [54]. Controlling inflammation through a given molecular route does not guarantee vascular benefit if that route carries its own thrombotic, oncologic or metabolic liability. IL-17 and IL-23 inhibitors appear to have at most modest short-term cardiovascular effects, with long-term outcomes still being defined [45].

General-population trials reinforce the same hierarchy from the opposite direction. Direct anti-inflammatory therapy targeting IL-1β with canakinumab reduced events, whereas low-dose methotrexate, which did not lower IL-6 or hsCRP in a non-rheumatic population, did not alter outcomes, which indicates that the relevant variable is the inflammatory pathway actually engaged rather than the drug class [5,11,12]. Statins, ezetimibe and proprotein convertase subtilisin/kexin type 9 (PCSK9) inhibitors remain indicated for lipid lowering, and bempedoic acid lowers both LDL-C and hsCRP, but disease-specific outcome trials in rheumatic populations are limited; the dedicated statin trial in RA, terminated early for a low event rate, showed the expected LDL-C and C-reactive protein reductions and a non-significant reduction in events consistent with the effect of statins in other populations [5,6,55]. The practical synthesis is that lipid-directed and inflammation-directed strategies are complementary rather than interchangeable, and that a rise in LDL-C after effective immunomodulation should be interpreted in the context of the inflammatory benefit rather than in isolation [7,8].

Table 3 consolidates the principal molecular mediators that connect chronic inflammation to vascular injury across the axes described above, together with the cellular site, biological consequence, main disease context and evidence category for each. It is intended as a map of the circuit at the level of molecules rather than as a comprehensive catalog. The final column explicitly distinguishes direct human rheumatic-disease evidence from general population, experimental or inferential support.

## 12. Clinical Translation and Cardiovascular Risk Stratification

The mechanistic argument has a direct corollary for risk assessment, but it should be stated cautiously. Conventional lipid panels can fail to capture lipoprotein dysfunction, and generic cardiovascular risk tools may underestimate absolute risk in autoimmune rheumatic disease, particularly during active inflammatory disease and in conditions where inflammation, glucocorticoid exposure, disease duration or disease-specific vascular mechanisms are not represented in the score [1,3,49]. This is not identical to saying that every model systematically underestimates risk in every patient or disease state. Rather, the same lipid concentration may carry different meaning depending on inflammatory activity and particle function. Proposed remedies include applying multipliers to standard scores, incorporating disease-specific factors, and adding biomarkers, although no rheumatic-disease-specific tool has been validated to replace generic algorithms [49].

The biomarkers that follow from the axes differ in how ready they are for clinical use, and that distinction should govern how they are deployed. ApoB and Lp(a) are clinically available measures that can complement standard cholesterol concentrations by reflecting atherogenic particle number and genetically anchored lipoprotein risk; Lp(a) may also be modulated by inflammatory signaling, including IL-6R blockade [9]. By contrast, cholesterol efflux capacity and other HDL functional assays are mechanistically informative but remain non-routine and insufficiently standardized for everyday cardiovascular risk prediction in rheumatology [16,20,21]. OxLDL measurements, inflammatory markers such as hsCRP and IL-6, and emerging lipidomic, metabolomic or proteomic signatures are promising research tools, but they should not yet be presented as validated clinical risk stratifiers. Vascular imaging adds information that bloodwork cannot, and carotid ultrasound, coronary artery calcium, flow-mediated dilation and pulse wave velocity can improve risk classification by detecting subclinical disease, although their implementation depends on guideline context, availability and individual patient risk [42,43].

Current guidance reflects this reasoning while acknowledging its limits. The relevant recommendations endorse regular screening and management of modifiable risk factors, advise that lipid management follow general-population guidelines, and make disease-specific points: hydroxychloroquine is recommended in SLE in part because it may reduce cardiovascular risk, a direction also supported by observational data in rheumatoid arthritis [51], glucocorticoid dose and disease activity should be minimized, and antiplatelet use should follow established profiles in SLE, APS and large-vessel vasculitis [49]. Several statements rest on expert opinion because high-quality evidence is lacking, which is itself a statement about the field. Effective prevention in these patients integrates lipid-lowering therapy, control of inflammatory disease activity, glucocorticoid minimization, attention to traditional risk factors and patient-specific modifiers, rather than any one of these in isolation [49].

## 13. Emerging Mechanisms

Several mechanisms sit at the edge of the current architecture and may prove load-bearing. Lp(a) deserves continued attention as a particle that is both genetically determined and inflammation-responsive, with oxidized phospholipids as a plausible mechanistic bridge to vascular risk in inflammatory states [9]. Adipose tissue is a further node: adipokines influence atherosclerosis in both protective and deleterious directions, and the obesity, insulin resistance and adipose inflammation that accompany psoriatic disease and other conditions interact with rheumatic inflammation and lipid dysfunction [44,56]. The gut microbiome is an emerging contributor, with dysbiosis linked to systemic inflammation and T helper 17 responses in RA through impaired barrier function and immune activation; its influence on lipid metabolism and vascular risk in rheumatic disease is plausible but, at present, largely inferential [57].

Other directions are noted because the evidence is thin rather than absent. Disease-specific lipidomic and metabolomic signatures, the contribution of remnant cholesterol and triglyceride-rich lipoproteins, the role of extracellular vesicles and microRNAs in lipid-immune signaling, and the influence of sex on lipid immunometabolism in diseases that disproportionately affect women are candidate mechanisms that the present architecture can accommodate but cannot yet incorporate with confidence. The most important unresolved question is quantitative rather than conceptual: not whether these mechanisms exist, but how much each contributes to events in a given patient, and whether targeting them changes outcomes.

Sex is a further dimension that the present architecture can frame but not yet quantify. Several autoimmune rheumatic diseases, most strikingly systemic lupus erythematosus, are markedly more common in women, and the determinants of cardiovascular risk in these patients plausibly include sex hormones and their effects on immune activation and lipid metabolism, pregnancy-related vascular exposures, the menopausal transition, the presence of antiphospholipid antibodies, cumulative glucocorticoid exposure, and undertreatment relative to men. These factors are individually plausible, but the evidence is not yet sufficient to support sex-specific lipid-immunometabolic risk algorithms. A practical step for the field is that future studies report sex-stratified analyses of lipoprotein function and cardiovascular outcomes wherever sample size allows, consistent with sex-and-gender reporting guidance.

## 14. Integrated Model

The axes assemble into a single explanatory model, but the model is evidence-weighted rather than uniform across diseases. Chronic immune activation can reorganize lipid metabolism so that the quantity of circulating lipoprotein and its function diverge. HDL may lose protective functions, ApoB particles may be oxidized or otherwise modified, and macrophage cholesterol handling may shift toward foam-cell formation and inflammasome activation. Beneath these events, acute immune-cell metabolic activation and, in some contexts, durable trained-immunity-like reprogramming may amplify cytokine output. The strongest direct human rheumatic-disease evidence comes from RA and SLE; cholesterol crystal-NLRP3 activation, trained immunity and some proposed feedback loops are supported mainly by general atherosclerosis or experimental data when applied to rheumatic disease. These streams converge on endothelial dysfunction and thrombo-inflammation, where modified lipoproteins, type I interferon, NETs, antiphospholipid antibodies and complement can contribute to atherosclerotic and thrombotic events.

Read this way, inflammation is the organizing variable, but not a universal substitute for lipid measurement. It can uncouple lipid concentration from risk through identifiable mechanisms, especially during active disease, yet the extent of this uncoupling differs by diagnosis, treatment status and vascular phenotype. The therapeutic evidence supports the hierarchy without making it absolute: controlling inflammation may reduce risk even when cholesterol rises, yet the route of control matters, because agents such as JAK inhibitors carry liabilities that can offset the benefit of suppressing inflammation. The model therefore points toward integrated assessment of particle number, lipoprotein function, inflammatory burden and subclinical vascular disease rather than replacement of conventional lipids by a single new biomarker.

## 15. Open Questions and Future Directions

Several questions follow directly from the architecture set out here and would, if answered, convert mechanistic plausibility into clinical utility. First, which mechanisms dominate in which disease and in which disease-activity state, given that the axes are weighted very differently across conditions. Second, whether direct measures of lipoprotein function, in particular cholesterol efflux capacity, can improve cardiovascular risk prediction beyond standard lipids in these populations, and whether they do so consistently across vascular beds. Third, which of the molecular mediators summarized here are actionable therapeutic targets rather than markers, and in which patients. Fourth, how lipid-lowering and anti-inflammatory strategies should be combined and sequenced, given that they are complementary rather than interchangeable and that the vascular liability differs by drug class. Fifth, whether lipidomic and metabolomic profiling can identify disease-specific risk signatures that conventional panels miss. Sixth, how sex, age, cumulative glucocorticoid exposure and treatment era modify the lipid-immune relationship. Seventh, whether disease-specific cardiovascular risk tools are feasible and worthwhile, or whether recalibrated general-population tools augmented by inflammatory and functional measures will suffice. The unifying theme is quantitative: the existence of these mechanisms is reasonably well established, but their relative contributions in the individual patient, and the outcome benefit of targeting them, remain to be defined.

## 16. Conclusions

Cardiovascular risk in autoimmune rheumatic diseases is best understood as a problem of lipid immunometabolism as well as lipid concentration. Systemic inflammation can reshape both how much lipoprotein circulates and how those particles behave, and the resulting dissociation, most visible as the lipid paradox in active rheumatoid arthritis, runs through HDL dysfunction, lipoprotein modification, macrophage foam-cell formation with inflammasome activation, immune-cell metabolic rewiring, and a convergent thrombo-inflammatory endothelium. The same architecture is not equally proven in every condition: RA and SLE provide the deepest mechanistic evidence, while for psoriatic disease, axial spondyloarthritis, systemic sclerosis, vasculitides and antiphospholipid syndrome the model rests on varying combinations of observational, imaging, surrogate, disease-specific or inferential evidence. The practical consequence is that conventional lipid panels and generic risk scores should not be interpreted in isolation, particularly during active inflammatory disease. Cardiovascular prevention should integrate lipid-lowering therapy, control of inflammatory activity, glucocorticoid minimization, traditional risk-factor management and attention to drug-specific vascular liabilities. The central open question remains quantitative: which mechanism dominates in which patient, and which targeted intervention will translate mechanistic plausibility into fewer events.

## Figures and Tables

**Table 1:** Disease-specific weighting of lipid-immune mechanisms, vascular phenotype, type of supporting evidence and principal uncertainty in autoimmune rheumatic diseases. Evidence categories C1-C6 are defined in Section 2.

Disease	Dominant Lipid-Immune Mechanism	Principal Mediators	Vascular Phenotype	Type of Evidence	Main Limitation/Uncertainty
Rheumatoid arthritis	Cytokine-driven lipoprotein remodeling; lipid paradox; impaired HDL function	IL-6, TNF-α, IL-1β; acute-phase response	Accelerated coronary atherosclerosis; excess MACE	C1 + C2: human outcome and mechanistic evidence (deepest)	Strongest evidence base, but mechanisms may not apply uniformly across treatment eras and disease-activity states
Systemic lupus erythematosus	Dysfunctional HDL; oxLDL and autoantibodies; interferon-driven endothelial injury	Type I interferon, immune complexes, complement, aPL	Premature atherosclerosis and thrombosis	C2 + C3: human mechanistic and surrogate/imaging evidence; outcome moderate	Heterogeneous outcome data; confounding by nephritis, glucocorticoids and coexisting APS
Psoriatic disease (PsA/psoriasis)	Inflammation amplifying metabolic-syndrome dyslipidemia	IL-17, IL-23, TNF-α; insulin resistance, adipokines	Increased CV events; metabolic comorbidity	C1 + C3: observational outcome plus surrogate evidence; metabolic confounding prominent	Strong cardiometabolic overlap, but separating inflammatory from obesity/metabolic-syndrome effects is difficult
Ankylosing spondylitis/axSpA	Inflammation-associated arterial stiffening; subclinical atherosclerosis	TNF-α, IL-17; systemic inflammation	Increased cIMT, arterial stiffness, reduced FMD	C3: surrogate/imaging evidence; limited hard-outcome or lipid-function evidence	Imaging evidence stronger than hard-outcome or mechanistic lipid evidence
Systemic sclerosis	Endothelial dysfunction; microvasculopathy with debated macrovascular extension	Endothelial injury, oxidative stress, fibrosis	Microvascular disease central; debated macrovascular atherosclerosis	C3 + C6: surrogate/imaging evidence plus mechanistic inference; macrovascular evidence contested	Macrovascular atherosclerosis remains debated; microvascular disease dominates
Vasculitis (large-vessel/ANCA-associated)	Vascular inflammation and endothelial injury; lipid contribution inferred	Cytokines, complement, NETs, endothelial activation	Ischemic heart, cerebrovascular and aortic disease (LVV); endothelial dysfunction (AAV)	C1 + C3 + C6: observational outcome and endothelial/surrogate evidence; lipid contribution inferred	Vascular inflammation is central, but lipid-immunometabolic mechanisms are less directly studied
Antiphospholipid syndrome	Atherothrombosis: oxLDL–β2GPI plus prothrombotic autoimmunity	aPL, β2GPI, complement, NETs, tissue factor	Arterial and venous thrombosis; accelerated atherosclerosis	C2 + C1: human mechanistic and observational evidence; disease-specific mechanism	Thrombo-inflammatory mechanisms are strong, but lipid pathways are disease-specific and not generalizable

aPL, antiphospholipid antibodies; axSpA, axial spondyloarthritis; cIMT, carotid intima-media thickness; CV, cardiovascular; FMD, flow-mediated dilation; HDL, high-density lipoprotein; MACE, major adverse cardiovascular events; NETs, neutrophil extracellular traps; oxLDL, oxidized low-density lipoprotein; PsA, psoriatic arthritis.

**Table 2:** Effects of antirheumatic and lipid-directed therapies on lipid pathways and cardiovascular risk, with interpretative caveats.

Therapy	Effect on Lipid Concentration	Effect on Lipoprotein Function/Inflammation	Net Cardiovascular Signal	Interpretative Caveat
Methotrexate (in RA)	Neutral to modest	Reduces systemic inflammation	Favorable in RA	Favorable observational RA signal, but non-RA CIRT trial neutral when IL-6/CRP not reduced
Hydroxychloroquine	Lowers LDL-C and total cholesterol	Anti-inflammatory; lipid-favorable	Recommended in SLE; may reduce risk	Favorable metabolic profile in SLE, but outcome evidence not definitive
Glucocorticoids	Raise lipids; adverse metabolism	Anti-inflammatory	Net adverse at higher cumulative dose	Anti-inflammatory benefit offset by cumulative cardiometabolic harm
TNF-α inhibitors	Variable; possible weight gain	Reduce vascular inflammation	Favorable signal in RA/PsA	Patient selection and disease-control confounding limit causal attribution
IL-6R inhibitors (tocilizumab)	Raise LDL-C, total cholesterol, triglycerides	Shift HDL toward anti-inflammatory; lower Lp(a), fibrinogen, D-dimer; raise paraoxonase	Surrogates improve; randomized MACE HR ~1.05 vs. etanercept	LDL-C rises while inflammatory/thrombotic surrogates improve; MACE HR vs. etanercept ~1.05 (~0.77–1.43); upper CI below prespecified non-inferiority margin, so large excess excluded and non-inferiority met, not proven cardioprotective
JAK inhibitors	Raise LDL-C and HDL-C	Control inflammation; thrombotic liability	ORAL Surveillance: MACE HR 1.33 (0.91–1.94); cancer HR 1.48 (1.04–2.09) vs. TNFi	Disease control did not ensure vascular safety; risk-enriched population; drug-specific liabilities
IL-17/IL-23 inhibitors	Limited change	Control Th17 inflammation	Modest; long-term uncertain	Long-term cardiovascular outcome evidence still limited
Statins/ezetimibe/PCSK9 inhibitors	Lower LDL-C	Statins lower hsCRP	Beneficial in general population	General-population outcome evidence strong; rheumatic disease-specific outcome evidence limited
Colchicine/IL-1β inhibition	Minimal lipid change	Suppress NLRP3–IL-1β–IL-6 axis	IL-1β neutralization reduced events (general population)	CANTOS (canakinumab, IL-1β) and colchicine trials show benefit in general atherosclerosis; benefit not directly established in autoimmune rheumatic populations

CV, cardiovascular; HDL, high-density lipoprotein; hsCRP, high-sensitivity C-reactive protein; LDL-C, low-density lipoprotein cholesterol; Lp(a), lipoprotein(a); MACE, major adverse cardiovascular events; PCSK9, proprotein convertase subtilisin/kexin type 9; PsA, psoriatic arthritis; RA, rheumatoid arthritis; SLE, systemic lupus erythematosus; TNFi, tumor necrosis factor inhibitor.

**Table 3:** Molecular mediators of lipid immunometabolism linking chronic inflammation to vascular injury. Evidence categories C1-C6 are defined in Section 2.

Pathway/Module	Key Molecular Mediators	Cellular Site	Biological Consequence	Main Disease Context	Evidence Level/Limitation
IL-6/acute-phase response	IL-6, JAK–STAT3, serum amyloid A, C-reactive protein, fibrinogen	Hepatocytes, immune cells	Acute-phase lipoprotein remodeling through hepatic IL-6 signaling; altered HDL cargo and inflammatory markers	RA, SLE	C4 supports IL-6 causality; C2 supports RA lipoprotein remodeling; outcome linkage indirect
TNF-α/inflammatory lipid remodeling	TNF-α, NF-κB	Hepatocytes, endothelium, macrophages	Altered lipoprotein production and clearance; endothelial activation	RA, PsA	Strong associative and treatment data; causal attribution limited by confounding
HDL remodeling	Serum amyloid A, paraoxonase-1, apolipoprotein A-I, LCAT, myeloperoxidase	HDL particle/plasma	Loss of antioxidant and anti-inflammatory HDL function; pro-inflammatory HDL	RA, SLE	Human mechanistic (strong); HDL function not a routine assay
Cholesterol efflux	ABCA1, ABCG1, apolipoprotein A-I/HDL acceptor	Macrophage membrane	Reverse cholesterol transport; efflux predicts events independent of HDL-C	General population; rheumatic (inferred)	Human cohort outcome data (general population); assay not standardized
Modified ApoB particles	oxLDL, oxidized phospholipids, neoepitopes, carbamylation	Subendothelial space, plasma	Foam-cell–substrate; autoantigen generation	RA, SLE, APS	oxLDL well supported; carbamylation–outcome link fragmentary
Scavenger-receptor uptake	CD36, SR-A, LOX-1; SR-BI mainly reflects HDL-associated bidirectional cholesterol flux	Macrophage	Modified-LDL uptake and foam-cell formation; CD36 nucleates intracellular crystals	General population; rheumatic (inferred)	C5 strong experimental; C6 for rheumatic-vessel extrapolation; SR-BI not equivalent to CD36/LOX-1
LXR–ABCA1/ABCG1 axis	Liver X receptors; ABCA1/ABCG1; TLR/NF-κB opposition	Macrophage	Transcriptional control of efflux; inflammation lowers efflux	General atherosclerosis	Experimental; mechanism extrapolated to rheumatic disease
NLRP3 priming (signal 1)	TLRs, NF-κB, NLRP3 induction, pro-IL-1β	Macrophage/myeloid	Licenses the inflammasome for activation	General population; rheumatic	Experimental; human proof via downstream IL-1β trials
Cholesterol-crystal NLRP3 activation (signal 2)	Cholesterol crystals, phagolysosomal rupture, caspase-1, IL-1β	Macrophage	Mature IL-1β release; IL-1β induces IL-6 in immune/stromal compartments; IL-6 drives hepatic acute-phase response	Atherosclerosis (rheumatic inferred)	C5 strong in experimental atherosclerosis; C6 when applied to rheumatic vessels
NETosis	NETs, PAD4, citrullinated autoantigens, myeloperoxidase	Neutrophils/low-density granulocytes	Endothelial injury; autoantigen exposure; macrophage priming	SLE, RA	C2 human mechanistic in SLE/RA; direct NET-mediated lipoprotein oxidation in vivo remains C6
Type I interferon endothelial injury	IFN-α/β, plasmacytoid dendritic cells	Endothelium, immune cells	Endothelial dysfunction; impaired repair	SLE	Human mechanistic; outcome link inferential
APS-specific oxLDL–β2GPI axis	oxLDL–β2GPI complexes, anti-β2GPI IgG, tissue factor	Macrophage, endothelium, platelets	Foam-cell formation; atherothrombosis	APS	Human mechanistic and observational; disease-specific
Immune-cell metabolic rewiring	AMPK, mTORC1, pentose phosphate pathway, HIF-1α, glycolysis, glutaminolysis	T cells, macrophages	Pro-inflammatory effector differentiation; sustained cytokine output	RA (T cells, direct); atherosclerosis (macrophage)	RA T-cell data direct; macrophage program partly extrapolated
Trained immunity/mevalonate pathway	Histone H3K4me3, mevalonate pathway, oxLDL, β-glucan	Myeloid progenitors, monocytes	Durable hyperinflammatory phenotype	Atherosclerosis (rheumatic inferred)	C5 strong in innate-immunity/atherosclerosis models; C6 for rheumatic CV outcomes

ABCA1/ABCG1, ATP-binding cassette transporters A1/G1; ApoB, apolipoprotein B; APS, antiphospholipid syndrome; CD36, cluster of differentiation 36; HDL, high-density lipoprotein; HDL-C, high-density lipoprotein cholesterol; HIF-1α, hypoxia-inducible factor 1-alpha; IFN, interferon; IL, interleukin; JAK, Janus kinase; LCAT, lecithin:cholesterol acyltransferase; LOX-1, lectin-like oxidized LDL receptor-1; LXR, liver X receptor; mTORC1, mechanistic target of rapamycin complex 1; NETs, neutrophil extracellular traps; NF-κB, nuclear factor kappa B; NLRP3, NLR family pyrin domain containing 3; oxLDL, oxidized low-density lipoprotein; PAD4, peptidylarginine deiminase 4; PsA, psoriatic arthritis; RA, rheumatoid arthritis; SLE, systemic lupus erythematosus; SR-A/SR-BI, scavenger-receptor A/B type I; STAT3, signal transducer and activator of transcription 3; TLR, Toll-like receptor; TNF-α, tumor necrosis factor-alpha; β2GPI, β2-glycoprotein I.

## Data Availability

No new data were created or analyzed in this study. Data sharing is not applicable to this article.

## References

[1] Venetsanopoulou A.I., Pelechas E., Voulgari P.V., Drosos A.A. (2020). The lipid paradox in rheumatoid arthritis: The dark horse of the augmented cardiovascular risk. Rheumatol. Int..

[2] Drosos A.A., Venetsanopoulou A.A., Pelechas E., Voulgari P.V. (2024). Exploring Cardiovascular Risk Factors and Atherosclerosis in Rheumatoid Arthritis. Eur. J. Intern. Med..

[3] Halacoglu J., Shea L.A. (2020). Cardiovascular Risk Assessment and Therapeutic Implications in Rheumatoid Arthritis. J. Cardiovasc. Transl. Res..

[4] Myasoedova E., Crowson C.S., Kremers H.M., Roger V.L., Fitz-Gibbon P.D., Therneau T.M., Gabriel S.E. (2011). Lipid paradox in rheumatoid arthritis: The impact of serum lipid measures and systemic inflammation on the risk of cardiovascular disease. Ann. Rheum. Dis..

[5] Libby P., Everett B.M. (2019). Novel Antiatherosclerotic Therapies. Arterioscler. Thromb. Vasc. Biol..

[6] Ridker P.M., Lei L., Louie M.J., Haddad T., Nicholls S.J., Lincoff A.M., Libby P., Nissen S.E. (2024). Inflammation and Cholesterol as Predictors of Cardiovascular Events Among 13,970 Contemporary High-Risk Patients With Statin Intolerance. Circulation.

[7] Barry S., Sheng E., Baker J.F. (2025). Metabolic Consequences of Rheumatoid Arthritis. Arthritis Care Res..

[8] Swerdlow D.I., Holmes M.V., Kuchenbaecker K.B., Engmann J.E.L., Shah T., Sofat R., Guo Y., Chung C., Peasey A., Interleukin-6 Receptor Mendelian Randomisation Analysis (IL6R MR) Consortium (2012). The interleukin-6 receptor as a target for prevention of coronary heart disease: A mendelian randomisation analysis. Lancet.

[9] McInnes I.B., Thompson L., Giles J.T., Bathon J.M., Salmon J.E., Beaulieu A.D., Codding C.E., Carlson T.H., Delles C., Lee J.S. (2015). Effect of interleukin-6 receptor blockade on surrogates of vascular risk in rheumatoid arthritis: MEASURE, a randomised, placebo-controlled study. Ann. Rheum. Dis..

[10] Reyes-Soffer G., Westerterp M. (2021). Beyond Lipoprotein(a) plasma measurements: Lipoprotein(a) and inflammation. Pharmacol. Res..

[11] Ridker P.M., Everett B.M., Thuren T., MacFadyen J.G., Chang W.H., Ballantyne C., Fonseca F., Nicolau J., Koenig W., Anker S.D. (2017). Antiinflammatory Therapy with Canakinumab for Atherosclerotic Disease. N. Engl. J. Med..

[12] Ridker P.M., Everett B.M., Pradhan A., MacFadyen J.G., Solomon D.H., Zaharris E., Mam V., Hasan A., Rosenberg Y., Iturriaga E. (2019). Low-Dose Methotrexate for the Prevention of Atherosclerotic Events. N. Engl. J. Med..

[13] Kamstrup P.R., Tybjaerg-Hansen A., Steffensen R., Nordestgaard B.G. (2009). Genetically elevated lipoprotein(a) and increased risk of myocardial infarction. JAMA.

[14] Ossoli A., Pavanello C., Giorgio E., Calabresi L., Gomaraschi M. (2019). Dysfunctional HDL as a Therapeutic Target for Atherosclerosis Prevention. Curr. Med. Chem..

[15] Ronda N., Zimetti F., Adorni M.P., Palumbo M., Karpouzas G.A., Bernini F. (2023). Role of Lipoprotein Levels and Function in Atherosclerosis Associated with Autoimmune Rheumatic Diseases. Rheum. Dis. Clin. North Am..

[16] Kim S.Y., Yu M., Morin E.E., Kang J., Kaplan M.J., Schwendeman A. (2020). High-Density Lipoprotein in Lupus: Disease Biomarkers and Potential Therapeutic Strategy. Arthritis Rheumatol..

[17] McMahon M., Grossman J., Skaggs B., FitzGerald J., Sahakian L., Ragavendra N., Charles-Schoeman C., Watson K., Wong W.K., Volkmann E. (2009). Dysfunctional proinflammatory high-density lipoproteins confer increased risk of atherosclerosis in women with systemic lupus erythematosus. Arthritis Rheum..

[18] Charles-Schoeman C., Watanabe J., Lee Y.Y., Furst D.E., Amjadi S., Elashoff D., Park G., McMahon M., Paulus H.E., Fogelman A.M. (2009). Abnormal function of high-density lipoprotein is associated with poor disease control and an altered protein cargo in rheumatoid arthritis. Arthritis Rheum..

[19] Tall A.R., Yvan-Charvet L. (2015). Cholesterol, inflammation and innate immunity. Nat. Rev. Immunol..

[20] Lee J.J., Chi G., Fitzgerald C., Kazmi S.H.A., Kalayci A., Korjian S., Duffy D., Shaunik A., Kingwell B., Yeh R.W. (2021). Cholesterol Efflux Capacity and Its Association With Adverse Cardiovascular Events: A Systematic Review and Meta-Analysis. Front. Cardiovasc. Med..

[21] Rohatgi A., Khera A., Berry J.D., Givens E.G., Ayers C.R., Wedin K.E., Neeland I.J., Yuhanna I.S., Rader D.R., de Lemos J.A. (2014). HDL cholesterol efflux capacity and incident cardiovascular events. N. Engl. J. Med..

[22] Shea S., Stein J.H., Jorgensen N.W., McClelland R.L., Tascau L., Shrager S., Heinecke J.W., Yvan-Charvet L., Tall A.R. (2019). Cholesterol Mass Efflux Capacity, Incident Cardiovascular Disease, and Progression of Carotid Plaque. Arterioscler. Thromb. Vasc. Biol..

[23] Frostegård J. (2023). Systemic lupus erythematosus and cardiovascular disease. J. Intern. Med..

[24] Zampieri S., Iaccarino L., Ghirardello A., Tarricone E., Arienti S., Sarzi-Puttini P., Gambari P., Doria A. (2005). Systemic lupus erythematosus, atherosclerosis, and autoantibodies. Ann. N. Y. Acad. Sci..

[25] Kobayashi K., Kishi M., Atsumi T., Bertolaccini M.L., Makino H., Sakairi N., Yamamoto I., Yasuda T., Khamashta M.A., Hughes G.R.V. (2003). Circulating oxidized LDL forms complexes with beta2-glycoprotein I: Implication as an atherogenic autoantigen. J. Lipid Res..

[26] Goel R.R., Kaplan M.J. (2020). Deadliest catch: Neutrophil extracellular traps in autoimmunity. Curr. Opin. Rheumatol..

[27] Khandpur R., Carmona-Rivera C., Vivekanandan-Giri A., Gizinski A., Yalavarthi S., Knight J.S., Friday S., Li S., Patel R.M., Subramanian V. (2013). NETs are a source of citrullinated autoantigens and stimulate inflammatory responses in rheumatoid arthritis. Sci. Transl. Med..

[28] Villanueva E., Yalavarthi S., Berthier C.C., Hodgin J.B., Khandpur R., Lin A.M., Rubin C.J., Zhao W., Olsen S.H., Klinker M. (2011). Netting neutrophils induce endothelial damage, infiltrate tissues, and expose immunostimulatory molecules in systemic lupus erythematosus. J. Immunol..

[29] Warnatsch A., Ioannou M., Wang Q., Papayannopoulos V. (2015). Neutrophil extracellular traps license macrophages for cytokine production in atherosclerosis. Science.

[30] Hong C., Tontonoz P. (2014). Liver X receptors in lipid metabolism: Opportunities for drug discovery. Nat. Rev. Drug Discov..

[31] Westerterp M., Fotakis P., Ouimet M., Bochem A.E., Zhang H., Molusky M.M., Wang W., Abramowicz S., la Bastide-van Gemert S., Wang N. (2018). Cholesterol Efflux Pathways Suppress Inflammasome Activation, NETosis, and Atherogenesis. Circulation.

[32] Tektonidou M.G. (2022). Cardiovascular disease risk in antiphospholipid syndrome: Thrombo-inflammation and atherothrombosis. J. Autoimmun..

[33] Duewell P., Kono H., Rayner K.J., Sirois C.M., Vladimer G., Bauernfeind F.G., Abela G.S., Franchi L., Nuñez G., Schnurr M. (2010). NLRP3 inflammasomes are required for atherogenesis and activated by cholesterol crystals. Nature.

[34] Grebe A., Latz E. (2013). Cholesterol crystals and inflammation. Curr. Rheumatol. Rep..

[35] Sheedy F.J., Grebe A., Rayner K.J., Kalantari P., Ramkhelawon B., Carpenter S.B., Becker C.E., Ediriweera H.N., Mullick A.E., Golenbock D.T. (2013). CD36 coordinates NLRP3 inflammasome activation by facilitating intracellular nucleation of soluble ligands into particulate ligands in sterile inflammation. Nat. Immunol..

[36] Grebe A., Hoss F., Latz E. (2018). NLRP3 Inflammasome and the IL-1 Pathway in Atherosclerosis. Circ. Res..

[37] Weyand C.M., Goronzy J.J. (2020). Immunometabolism in the development of rheumatoid arthritis. Immunol. Rev..

[38] Christ A., Günther P., Lauterbach M.A., Duewell P., Biswas D., Pelka K., Scholz C.J., Oosting M., Haendler K., Baßler K. (2018). Western Diet Triggers NLRP3-Dependent Innate Immune Reprogramming. Cell.

[39] Bekkering S., Quintin J., Joosten L.A.B., van der Meer J.W.M., Netea M.G., Riksen N.P. (2014). Oxidized low-density lipoprotein induces long-term proinflammatory cytokine production and foam cell formation via epigenetic reprogramming of monocytes. Arterioscler. Thromb. Vasc. Biol..

[40] Tercan H., Riksen N.P., Joosten L.A.B., Netea M.G., Bekkering S. (2021). Trained Immunity: Long-Term Adaptation in Innate Immune Responses. Arterioscler. Thromb. Vasc. Biol..

[41] Ambler W.G., Kaplan M.J. (2024). Vascular damage in systemic lupus erythematosus. Nat. Rev. Nephrol..

[42] Bai R., Zhang Y., Liu W., Ma C., Chen X., Yang J., Sun D. (2019). The Relationship of Ankylosing Spondylitis and Subclinical Atherosclerosis: A Systematic Review and Meta-Analysis. Angiology.

[43] Bertolino J., Scafi M., Benyamine A., Aissi K., Boufi M., Schleinitz N., Sarlon G., Rossi P., Granel B. (2019). Systemic sclerosis and macrovascular involvement: Status of the issue in 2019. J. Med. Vasc..

[44] Toussirot E., Gallais-Sérézal I., Aubin F. (2022). The cardiometabolic conditions of psoriatic disease. Front. Immunol..

[45] Williams J.C., Hum R.M., Rogers K., Maglio C., Alam U., Zhao S.S. (2024). Metabolic syndrome and psoriatic arthritis: The role of weight loss as a disease-modifying therapy. Ther. Adv. Musculoskelet. Dis..

[46] Clifford A.H. (2023). Cardiovascular Disease in Large Vessel Vasculitis: Risks, Controversies, and Management Strategies. Rheum. Dis. Clin. North Am..

[47] Massicotte-Azarniouch D., Petrcich W., Walsh M., Canney M., Hundemer G.L., Milman N., Hladunewich M.A., Fairhead T., Sood M.M. (2022). Association of anti-neutrophil cytoplasmic antibody-associated vasculitis and cardiovascular events: A population-based cohort study. Clin. Kidney J..

[48] Erdbrügger U., Kielstein J.T., Westman K., Ma J.Z., Xin W., Bode-Böger S.M., Segelmark M., Rasmussen N., De Groot K. (2018). Higher levels of SDMA and not ADMA are associated with poorer survival of trial patients with systemic ANCA-associated vasculitis. Eur. J. Rheumatol..

[49] Drosos G.C., Vedder D., Houben E., Boekel L., Atzeni F., Badreh S., Boumpas D.T., Brodin N., Bruce I.N., González-Gay M.Á. (2022). EULAR recommendations for cardiovascular risk management in rheumatic and musculoskeletal diseases, including systemic lupus erythematosus and antiphospholipid syndrome. Ann. Rheum. Dis..

[50] Giles J.T., Sattar N., Gabriel S., Ridker P.M., Gay S., Warne C., Musselman D., Brockwell L., Shittu E., Klearman M. (2020). Cardiovascular Safety of Tocilizumab Versus Etanercept in Rheumatoid Arthritis: A Randomized Controlled Trial. Arthritis Rheumatol..

[51] Sharma T.S., Wasko M.C.M., Tang X., Vedamurthy D., Yan X., Cote J., Bili A. (2016). Hydroxychloroquine use is associated with decreased incident cardiovascular events in rheumatoid arthritis patients. J. Am. Heart Assoc..

[52] Roubille C., Richer V., Starnino T., McCourt C., McFarlane A., Fleming P., Siu S., Kraft J., Lynde C., Pope J. (2015). The effects of tumour necrosis factor inhibitors, methotrexate, non-steroidal anti-inflammatory drugs and corticosteroids on cardiovascular events in rheumatoid arthritis, psoriasis and psoriatic arthritis: A systematic review and meta-analysis. Ann. Rheum. Dis..

[53] Ytterberg S.R., Bhatt D.L., Mikuls T.R., Koch G.G., Fleischmann R., Rivas J.L., Germino R., Menon S., Sun Y., Wang C. (2022). Cardiovascular and Cancer Risk with Tofacitinib in Rheumatoid Arthritis. N. Engl. J. Med..

[54] Smolen J.S., Landewé R.B.M., Bergstra S.A., Kerschbaumer A., Sepriano A., Aletaha D., Caporali R., Edwards C.J., Hyrich K.L., Pope J.E. (2023). EULAR recommendations for the management of rheumatoid arthritis with synthetic and biological disease-modifying antirheumatic drugs: 2022 update. Ann. Rheum. Dis..

[55] Kitas G.D., Nightingale P., Armitage J., Sattar N., Belch J.J.F., Symmons D.P.M., TRACE RA Consortium (2019). A Multicenter, Randomized, Placebo-Controlled Trial of Atorvastatin for the Primary Prevention of Cardiovascular Events in Patients With Rheumatoid Arthritis. Arthritis Rheumatol..

[56] Liu L., Shi Z., Ji X., Zhang W., Luan J., Zahr T., Qiang L. (2022). Adipokines, adiposity, and atherosclerosis. Cell. Mol. Life Sci..

[57] Zhao T., Wei Y., Zhu Y., Xie Z., Hai Q., Li Z., Qin D. (2022). Gut microbiota and rheumatoid arthritis: From pathogenesis to novel therapeutic opportunities. Front. Immunol..

